# Replication termination without a replication fork trap

**DOI:** 10.1038/s41598-019-43795-2

**Published:** 2019-06-05

**Authors:** Elisa Galli, Jean-Luc Ferat, Jean-Michel Desfontaines, Marie-Eve Val, Ole Skovgaard, François-Xavier Barre, Christophe Possoz

**Affiliations:** 1 Institute for Integrative Biology of the Cell (I2BC), Université Paris-Saclay, CEA, CNRS, Université Paris Sud, Gif sur Yvette, France; 2 Université de Versailles-Saint-Quentin, Versailles, France; 3 Bacterial Genome Plasticity, Genomes & Genetics Department, Institut Pasteur, Paris, 75015 France; 4 UMR3525, Centre National de la Recherche Scientifique, Paris, 75015 France; 5 Department of Science, Systems and Models, Roskilde University, Roskilde, 4000 Denmark

**Keywords:** Prokaryote, Bacterial evolution

## Abstract

Bacterial chromosomes harbour a unique origin of bidirectional replication, *oriC*. They are almost always circular, with replication terminating in a region diametrically opposite to *oriC*, the terminus. The *oriC*-terminus organisation is reflected by the orientation of the genes and by the disposition of DNA-binding protein motifs implicated in the coordination of chromosome replication and segregation with cell division. Correspondingly, the *E. coli* and *B. subtilis* model bacteria possess a replication fork trap system, Tus/*ter* and RTP/*ter*, respectively, which enforces replication termination in the terminus region. Here, we show that *tus* and *rtp* are restricted to four clades of bacteria, suggesting that *tus* was recently domesticated from a plasmid gene. We further demonstrate that there is no replication fork system in *Vibrio cholerae*, a bacterium closely related to *E. coli*. Marker frequency analysis showed that replication forks originating from ectopic origins were not blocked in the terminus region of either of the two *V. cholerae* chromosomes, but progressed normally until they encountered an opposite fork. As expected, termination synchrony of the two chromosomes is disrupted by these ectopic origins. Finally, we show that premature completion of the primary chromosome replication did not modify the choreography of segregation of its terminus region.

## Introduction

Genome architecture is extremely conserved in bacteria. Bacterial genomes are typically organised on a single chromosome, which rarely exceeds 10 Mbp and which is almost always circular^1^. Bacterial replication is initiated bidirectionally at a unique origin of replication per chromosome, *oriC*. Provided that they progress at a similar velocity, two replication forks originating from *oriC* converge in a zone diametrically opposite to it, which is called the terminus (Ter). Completion of replication at a precise place and time seems to participate in the coordination of the different cellular processes: replication, segregation and cell division^2^. It was notably illustrated by the observation that the termini of the different circular chromosomes harboured by bacteria with a multipartite genome, such as *Vibrio cholerae*, are replicated synchronously at mid-cell^3–7^. The system allowing the synchrony of termination of the two chromosome of *V. cholerae* is based on the fact that the secondary chromosome (chr2) initiates replication only when *crtS*, a non-coding locus located 690 kb downstream of *oriC1*, between VC764 and VC765 genes, on the primary chromosome (chr1) has been replicated^8–11^.

Ter is functionally defined via hundreds of small strategically-located DNA motifs^2,12^. First, FtsK-Orienting Polar Sequences (KOPS) and SpoIIIE-Recogntition Sequences (SRS) are distributed all over the unique chromosome of *E. coli* and *B. subtillis* and over the two chromosomes of *V. cholerae*. KOPS and SRS are skewed octameric motifs directing DNA transaction towards the Ter region, which contributes to the completion of chromosome segregation^13–18^. They notably bring together sister copies of a highly conserved 28 bp motif located in the middle of the Ter region, *dif*, which contributes to sister chromosome decatenation and enable the resolution of chromosome dimers^19–23^. Second, *E. coli*, *V. cholerae* and *B. subtilis* contain an inhibitor of cell division, either SlmA or Noc, which prevents the assembly/ stabilisation of a septum ring over the bulk of the nucleoid. SlmA and Noc bind to specific motifs, which are located all over the chromosomes but excluded from the Ter. As a result, septum formation only occurs when chromosomes have been segregated and is precisely located at mid-cell where the Ter is maintained^24–28^. Third, MatP, which binds to specific DNA motifs in the Ter region, contributes to the coordination of cell division and chromosome segregation by maintaining the two sister copies of the terminus regions together at mid-cell at the time of division in *E. coli* and in *V. cholerae*^29–31^. In *E. coli*, the MatP/*matS* complexes also stabilises the FtsZ-ring assembly *via* its direct interaction with the cell division protein ZapB^31,32^. Hence, the coordination between chromosome segregation and cell division benefits from the completion of DNA synthesis at Ter.

In the absence of replication problems, the two independent replication forks originating from the unique origin of bacterial circular chromosomes naturally converge in the Ter region^33,34^. However, several challenging situations require a replication fork trap (RFT) system to force termination within the Ter region. Replication initiated at ectopic positions such as those occupied by prophages or those resulting from proposed over-replication are a few examples^35^. Cells may also benefit from an RFT preventing a replication fork to progress on the illegitimate replichore if the other fork is prematurely halted or inactivated^36,37^. The drastic loss of viability of mutant cells unable to reactivate replication forks in *E. coli* suggests that the occurrence of such fork impediments is not rare, especially in rich medium^38^. Protein-DNA complexes are a major source of replication fork pausing in *E. coli*^39^. RFT may also circumvent deleterious head-to-head collisions occurring between replication forks and transcription bubbles by preventing replication forks to proceed replication beyond Ter. Indeed, the transcription of highly expressed genes on each replichore is oriented in the direction of the replication fork, which is supposed to ensure a smooth and processive synthesis of DNA^40^.

Two unrelated RFT systems were described so far, *ter*/Tus in *E. coli* and *ter*/ RTP in *B. subtilis*^41–43^. Both systems are based on two types of components and operate similarly. Surrounding the Ter region, several copies of *ter*-sites, which are non-palindromic sequences, point toward the *dif* site. The Tus or RTP proteins bind in an oriented manner on these *ter*-sites in such a way that only the forks progressing from, but not towards, *dif* are blocked^44,45^. The efficiency of blockage depends on each *ter* site^46,47^ and on the replication fork speed^48^. In laboratory conditions, inactivation of these systems does not generate strong phenotypes^42^. However, the fitness is slightly reduced in *E. coli tus* mutant cells containing an additional ectopic replication origin (*oriZ*): *oriC*-*oriZ tus* cells have a doubling time of 21.6 min compared to the 20.5 min doubling time of *oriC*–*oriZ* cells. These observations suggested that completion of replication within the Ter region participated in the processivity of replication fork progression^49^. The RFT effect on replication fork progression was monitored in *E. coli* using Marker Frequency Analysis (MFA) in strains harbouring an additional ectopic origin: in presence of an RFT, the convergence between the two forks continued to be in the Ter region, while in its absence (*tus* mutant), the convergence point was displaced to the midpoint between the two origins^36,49^. However, no homologous copy of the *E. coli tus* or the *B. subtilis rtp* genes was found in its genome with a BLAST-P search, even though *V. cholerae* contains homologues of all the other elements differentiating Ter from the rest of the chromosome in *E. coli*, including SlmA-binding sites, *matS*, KOPS and *dif*.

Here, we investigated the possible existence of a replication fork trap (RFT) in *V. cholera*e. We confirmed using a Hidden-Markov Model (HMM) approach that *V. cholerae* possessed neither *tus* nor *rtp*. Our phylogenetic observations further revealed that “resident” *tus* and *rtp* are limited to a narrow range of bacteria, which suggests that *tus* was recently domesticated from plasmid genes. To verify whether *V. cholerae* uses another yet unknown RFT system or not, we directly analysed the positions where forks converged in strains harbouring ectopic origins of replication. Our results demonstrate that there is no RFT on either of the two *V. cholerae* chromosomes. Finally, we show that the coordination between the segregation of the primary chromosome and cell division is not significantly impaired when Ter is not the last region to be replicated.

## Results

### *tus* and *rtp* are absent in the Vibrionales

**Figure 1:**
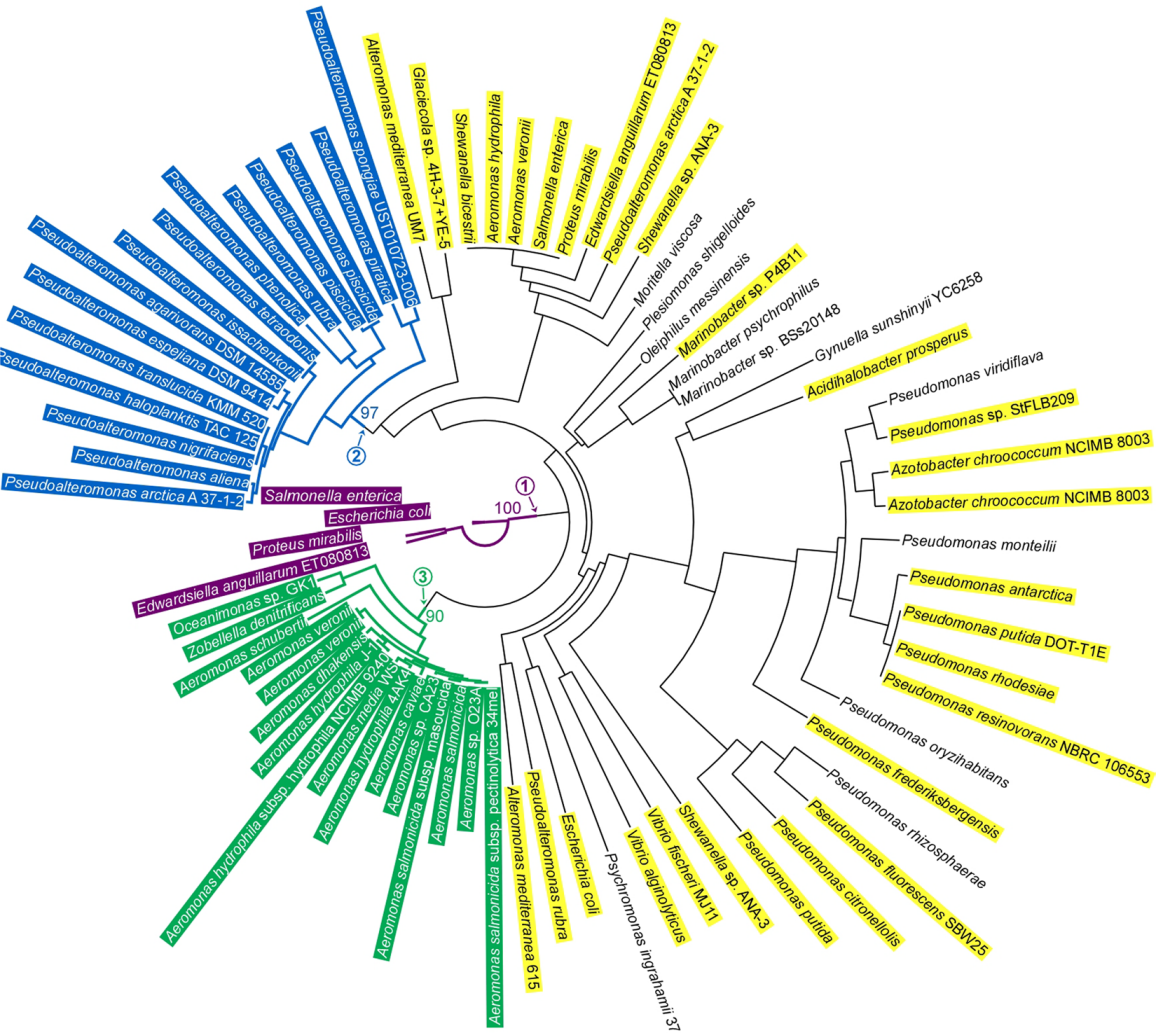
Tus-related homologs in bacteria. Phylogenetic tree of the proteins containing the Tus HHM profile (PF05472) found in complete genome of UniProt database and named as the sequenced species. Only three monophyletic groups of proteins were considered as “resident” as they present a distribution congruent with that of the species containing these Tus proteins established with a DnaABEX-based phylogenetic tree. Most Enterobacteriales (only a subset is indicated) highlighted in purple ①, the *Pseudoalteromonas* in which *tus* is present on the second chromosome highlighted in blue ②, and all the Aeromonadales, except *Tolumonas aurensis* TA4, highlighted in green ③. By opposition, the other *tus*-related genes, which do not meet the residency criteria explained in the main text, are considered as “mobile” genes. Some are plasmid-borne (highlighted in yellow) and other are located on chromosomes (not highlighted). Scale bar represents 0.1 substitutions per site; Bootstrap scores of domestication branches are indicated on the figure.

We searched the UniProt database for the presence of protein sequences containing the Tus (PF05472) or the RTP (PF02334) HMM profiles. No RTP-related sequences were found outside of a subgroup of Bacillales (data not shown). Similarly, we identified *tus* genes only among γ-proteobacteriales strains (Fig. 1). Some *tus* genes were detected in the Vibrionales, like *V. cholerae*. A fraction is plasmid-borne while other, located on the chromosome, appears to be distributed erratically among the Vibrionales. This led us to define which among the *tus* genes are “resident” or “mobile” (see Supp. Information).

We identified three monophyletic groups of Tus proteins. The distribution of the Tus protein within each of these three groups is congruent with the phylogenetic tree based on the DnaABEX proteins of the species containing these Tus proteins. In addition, the genomic context of theses *tus* gene is conserved among each group. From these two criteria, we define these *tus* gene as resident. The three groups with a resident *tus* are: most Enterobacteriales (only a subset is indicated) highlighted in purple ①, the Pseudoalteromona*s* in which *tus* is present on the second chromosome highlighted in blue ②, and all the Aeromonadales, except *Tolumonas aurensis* TA4, highlighted in green ③ (Fig. 1). The other identified *tus* genes do not fulfil these criteria and were then defined as “mobile”. Their genomic location is not conserved and their distribution is not congruent with that of the species in which they were found. Indeed, they are frequently plasmid-borne (highlighted in yellow, Fig. 1).

The proposed position of *tus* domestication in the three distinct clades was deduced from the distribution of resident *tus*. The vast majority of Enterobacteriales contains *tus* (highlighted in purple, Fig. S1) and an analysis of the *tus* genomic context reveals that it is strictly conserved (data not shown). A monophyletic group of species, containing mostly endosymbionts, might have lost *tus* in the course of evolution. Finally, we noticed that *tus* is absent in *Plesiomonas shigelloides*, a bacterium that split early in the Enterobacteriales (Fig. S1). Altogether, these observations suggest that *tus* was acquired and domesticated shortly after the origination of the Enterobacteriales (①, Figs 1, S1). The second clade containing *tus* regroups the Pseudoaleromonadacae (②, Figs 1, S1). These species are atypical Alteromonadales since they contain a second chromosome of plasmid origin. Within the Pseudoaleromonadacae, *tus* is strictly and systematically associated with the secondary chromosome, suggesting that the gene became resident during the domestication of the plasmid into a chromosome. Interestingly, in the Pseudoaleromonadacae *tus* is systematically located next to the origin of replication. Additionally, *tus* was identified within all Aeromonadales except *Tolumonas aurensis* TA4 where it might have been lost (③, Figs 1, S1). We conclude from this phylogenomic analysis that there is neither resident *tus* nor *rtp* in Vibrionales and in most bacterial orders.

### A marker frequency analysis method to define fork convergence points

**Figure 2:**
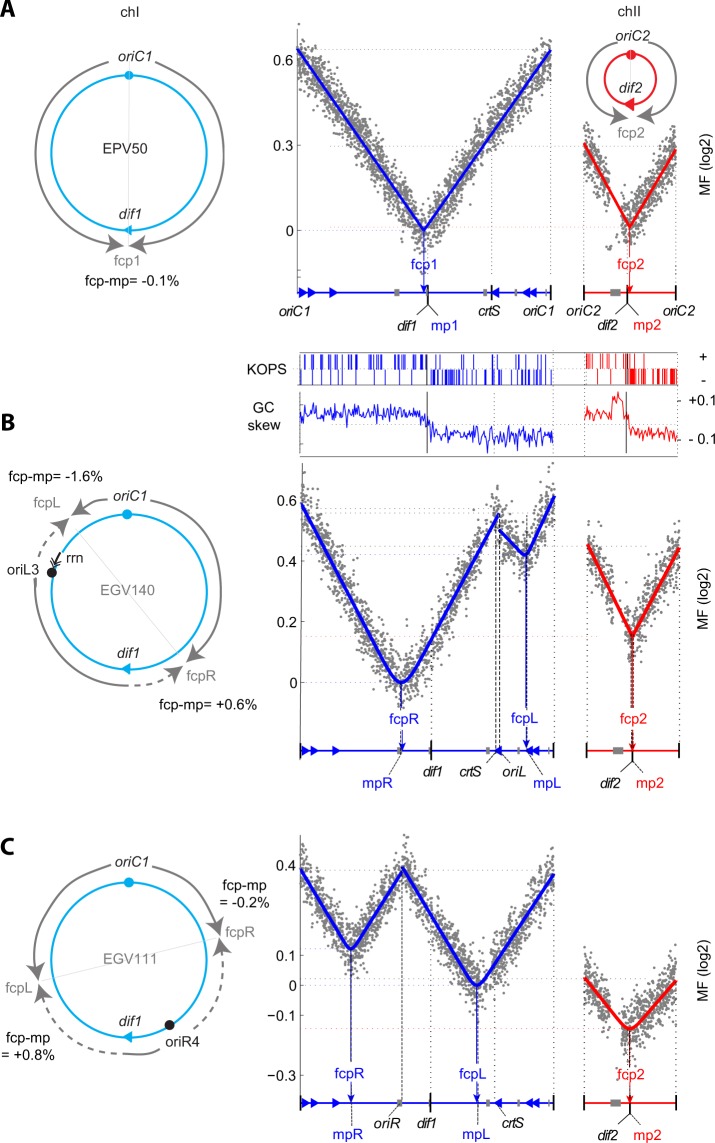
MFA of the two-chromosome strains: *V. cholerae* N16961 (EPV50; **A**) and derivatives containing ectopic *oriC1* ((EGV140; (**B**) and EGV111; **C**)). Marker frequencies (grey dots after trimming (see Supp. Methods) and normalisation on the total number of reads) are represented in Log2 as a function of the genome position. The *oriC1* or *oriC2* of chr1 or chr2, respectively, are indicated at each extremity. Position of *dif1*, *dif2*, ectopic origin if applicable (*oriL3* or *oriR4*), *crtS*, the different mp (origins mid-point) are indicated. The lowest point on chr1 was set to “1” in such a way that log2(1) = “0” and all data were normalized to this point. The curve fitting the marker frequency data (see Supp. Methods for MFA method) are indicated by either a blue or a red line for chr1 and chr2, respectively. They define the forks convergence points (fcp), indicated under the data. On the left side of the marker frequency data, a scheme representing the program of replication of chr1 is indicated on the circular map of the different strains. The program of replication of chr2 is represented only for EPV50 as it is not modified in EGV140 and EGV111. The program of replication deduced from the MFA is as follow: plain grey line corresponds to the wild-type direction of fork progression and the dashed grey line to the reverse direction of fork progression. The distance between fcp and its mp (noted fcp-mp) is indicated in % of the replicon fraction, oriented from the first origin encounters in the clock-wise direction.

**Figure 3:**
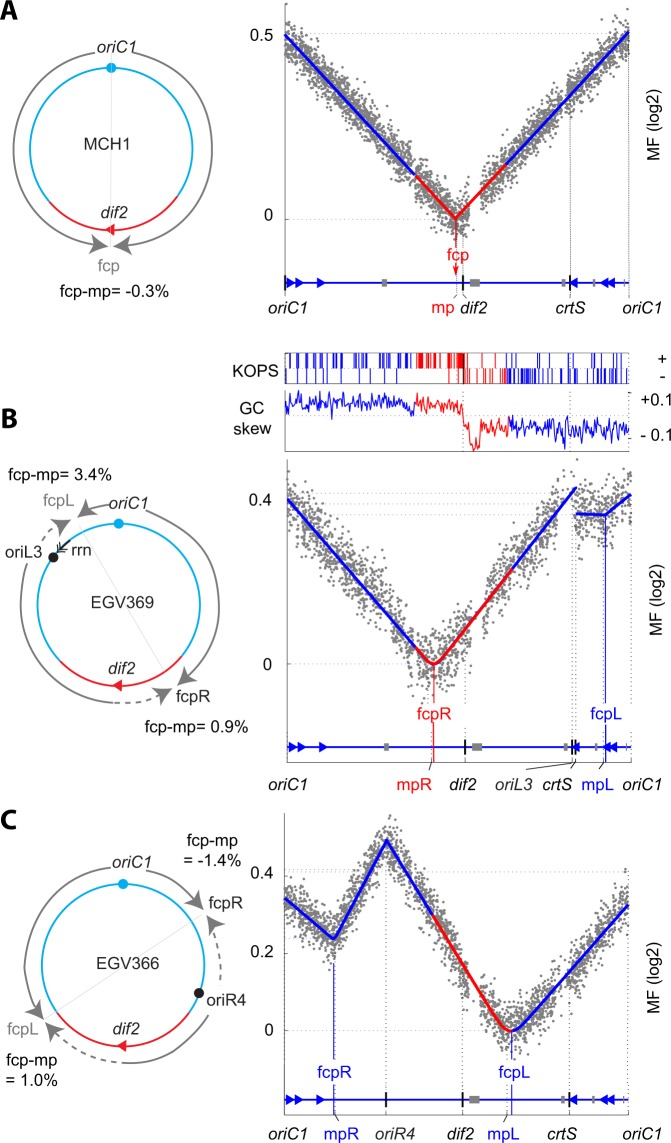
MFA of the mono-chromosome strains: *V. cholerae* MCH1 (**A**) and derivatives containing ectopic *oriC1* (EGV369; (**B**) and EGV366; (**C**)). See Fig. 2 legend, adapted for the fusion between chr1 and chr2 (deleting *dif1* region and *oriC2* region).

In exponentially growing cells, forks originating from the same origin of replication progress at similar speed and converge in a region precisely opposite to the origin. In other word, the fork convergence point (fcp) corresponds to the midpoint of the fragment replicated (mp). It is noteworhy that, using replication-synchronised conditions, the two sides of the *E. coli oriC* or of the *V. cholerae oriC1* origins were shown to present an asymmetry of replication start leading to displacement of the expected fcp from the mp^7,33^. However, we could not observe any displacement in our data of WT situations (Figs 2, 3). In contrast, because of the presence of the RFT in *E. coli*, the fcp may significantly differ from the mp when the origin of replication is displaced or when an extra origin of replication is added^36,49,50^. Hence, this fcp, displaced from mp, would indicate the position of the RFT. Therefore, we decided to check whether the addition of extra-origins of replication could displace the fcp away from the mp on the two *V. cholerae* chromosomes. Without RFT, the coincidence between mp (midpoint between the two origins) and fcp is expected if the ectopic origins initiate replication synchronously with the endogenous origin. To this end, we developed an MFA method to quantify the deviation of the fcp of any two converging forks from the expected mp. For all the strains we studied, the marker frequencies decreased exponentially between the replication origins and their cognate fcp, as expected from the Cooper-Helmstetter model of replication (see Figs 2, 3;^51^). We could thus calculate the fcp between any two origins as the position that minimised the error between the log of the experimental marker frequency values and the theoretic values given by their linear regression (see Supp. Methods). In the case of cells carrying ectopic origins of replication, we noticed a slight deviation of the log of the experimental marker frequencies from the linear regression model around the calculated fcp. Since the fcp derived from MFA is the average position where replication forks converged in a population of exponentially growing cells, we corrected the model by considering that the frequency of the cells in which forks converged at any specific position formed a gaussian centred on fcp. The width of the gaussian minimising the error between the experimental data and the gaussian-corrected model indicates the size of the region centred on the fcp within which 95% of the forks converged, which we termed S95 (see Supp. Methods). In order to validate our MFA method, we used it to define the origin regions of our WT strains. In both replicates and using different sliding window sizes, we could localise the position of “origin” at less than 0.5% from the real *oriC1* (see Supp. Methods).

### *V. cholerae* chr1 lacks an RFT

We first applied our MFA method to EPV50, an El Tor N16961 *V. cholerae* strain, to EGV140, a derivative harbouring an additional copy of the origin of replication of chr1 (*oriC1*) at 0.650 Mbp (position called L3 previously^52^) from *oriC*1 on the left replichore of chr1 (*oriL3*) and to EGV111, a derivative harbouring an additional copy of *oriC1* at 1.190 Mpb (position called R4 previously^52^) from *oriC1* on the right replichore (*oriR4*). The presence of the ectopic origins did not affect considerably the generation time in rich and minimal medium (Table S2).

The MFA of exponentially growing EPV50 cells in minimal medium showed that the fcp of chr1, fcp1, was only ~45 kb away from *dif1* and deviated from the mp of the replicon, mp1, by only 0.1% of the replicon length (Fig. 2A and Table S4). The MFA of exponentially growing EGV140 and EGV111 cells in minimal medium presented a drastically modified chr1 profile (Fig. 2B,C). Consistent with synchronous initiation of replication at the two origins of chr1, the marker frequencies of the *oriC1* and *oriL3* loci were the highest and were roughly identical (Fig. 2B and Table S2). The fcp of the right replicon (fcpR) was moved ~320 kb away from fcp1 (Fig. 2B and Table S4). It was only at 14 kbp from the midpoint of the right replicon, mpR, which corresponds to a deviation of 0.6% of the replicon length (Fig. 2B and Table S4). The fcp observed on the left replicon, fcpL, was about 9 kb from the midpoint of the replicon, mpL, corresponding to a 1.6% deviation (Fig. 2B and Table S4). Similarly, replication was initiated synchronously at the two chr1 origins of EGV111, *oriC1* and *oriR4* (Fig. 2C and Table S2). The fcp of the right replicon, fcpR, was only at 1 kbp from the midpoint of this replicon, mpR, corresponding to a 0.2% deviation (Fig. 2C and Table S4). The fcp of the left replicon, fcpL, was at 9 kbp from the midpoint of the replicon, mpL, corresponding to a 0.8% deviation (Fig. 2C and Table S4). Thus, a replication fork originating from *oriR4* can progress normally past fcp1 and *dif1* over 585 kb up to fcpL of EGV111, corresponding to ~40% of the left replichore. A replication fork can also progress over 810 kb without perturbation from *oriR4* toward the fcpR of EGV111. With the addition to the 320 kb already tested in EGV140, it corresponds to a total of ~60% of the right replichore (Fig. 2B,C). Taken together, these results demonstrate that there is no RFT on chr1.

### Additional origins of replication affect termination synchrony of chr1 and chr2

The MFA of exponentially growing EPV50 cells showed that the fcp of chr2, fcp2, was only ~30 kbp away from *dif2* and deviated from the mp of the replicon, mp2, by only 0.04% of the replicon length (Fig. 2A and Table S4). The presence of *oriL3* and *oriR4* on chr1 did not affect significantly the fcp on chr2 (Fig. 2B,C and Table S4). However, it perturbed the synchrony of termination of chr1 and chr2. The synchrony of termination of the two chromosomes is due to a time-delay between the initiation of chr1 and chr2 replication, chr2 replication initiation requiring duplication of a specific DNA motif adequately located on chr1, *crtS*^7–9,53^.

In EGV140, the *crtS* locus is located at about 45 kb from *oriL3*. It is replicated soon after chr1 replication initiation and in agreement with the function of *crtS*, chr2 replication is initiated early and the marker frequency of *oriC2* is quite similar to the marker frequency *oriL3* (Fig. 2B and Table S2). As a result, the marker frequency of fcp2 is significantly higher than the marker frequency of the fcpR indicating that chr2 replication terminates ahead of chr1 replication (Figs 2B, S4 and Table S2). Reciprocally, in the strain containing *oriR4*, the replication of *crtS* is delayed since the sequence is located close to fcpL, the last region of chr1 to be replicated, and the marker frequency of *oriC2* is similar to the marker frequency of fcpL (Fig. 2C and Table S2). As result, chr2 replication terminates well after chr1 has been replicated (Figs 2C, S4 and Table S2).

### Progression on an illegitimate replichore slightly perturbs replication

In EGV140, the fcp of the left replicon, fcpL, was shifted 9 kbp closer to the ectopic origin than expected at position mpL. In addition, we noticed that the marker frequencies immediately on the left and right of *oriL3* and the slopes of the *oriL3*-fcpL and *oriL3*-fcpR regression lines were markedly different (Fig. 2B and Table S2). A likely explanation for these observations was the presence of a ribosomal RNA genes (*rrn)* operon in *oriL3*-fcpL replichore, which is transcribed in a direction opposite to that of the replication (Fig. 2B, double arrow head). Indeed, previous work indicated that head-on collisions between replication forks and transcription bubbles could result in fork stalling and/or DNA degradation-dependent repair^37^. In agreement with this hypothesis, marker frequencies were significantly lower on the right of *oriL3* than its left in fast growing cells where the transcription of the *rrn* operon is expected to be stronger (Figs S5, S6 and Table S2). Moreover, the fcpL was closer to *oriL3*, now deviating from mpL by 11.4% (Fig. S6 and Table S4). No such dramatic deviation of the fcp from the mp was observed for the right replicon of EGV140 and the two replicons of EGV111 (Fig. 2B,C, Table S4). However, under fast growth conditions, the fcp was always situated on the side of the mp that minimised replication on illegitimate replichores (Figs S5–S7 and Table S4). In addition, S95, the length of the region centred on the fcp within which 95% of forks converged, increased from 4 kbp for the fcp1 of EPV50 to 128 kbp for the fcpL of EGV140, 628 kpb for the fcpR of EGV140, 396 kbp for the fcpL of EGV111 and 176 kbp for the fcpR of EGV111 (Fig. 2 and Table S4). Together, these results suggest that replication is slightly perturbed when ectopic origins were added.

### *V. cholerae* chr2 lacks an RFT

The Ter of chr2 (Ter2) appears to contain all the attributes of Ter1 such as the underrepresentation of the SlmA-binding sites, the presence of *matS* sites, the convergence of the KOPS toward a *dif* site^20,24,29,30^. To investigate the presence of an RFT on chr2, we added an additional ectopic origin at two positions in a mono-chromosomal *V. cholerae* strain, MCH1^54^, in which the two replicons are fused to each other through the replacement of the *dif1* region of chr1 by the entire sequence of chr2, excepting the *oriC2* and partition machinery region (Fig. 3A). The MFA of MCH1 showed that replication initiated at *oriC1* and that replication forks progressed across chr1 and chr2 DNA to converge close to *dif*2. The fcp was at 13 kb away from the midpoint, corresponding to a deviation of ~0.3% of the replicated DNA segment. Marker frequency slopes decreased exponentially at the same rate on each replication arm (Fig. 3A).

The marker frequency profiles of the MCH1 strains containing *oriL3* (EGV369) or *oriR4* (EGV366) indicated that replication initiated at both the native and the ectopic origin. In EGV369, we noticed a reduction in marker frequency associated with the right replication fork, emerging from *oriL3*, which is probably caused by the transcription of the *rrnE* operon. The marker frequencies were noisy and we did not trust any fitting over the corresponding replicon. However, it did not prevent the analysis of the other replicon of EGV369. Likewise, the unexpectedly lower marker frequency at *oriC1* than at *oriR4* in EGV366 did not prevent the analysis of the fcp of the two replicons. In EGV369, the fcpR was located 360 kb away from *dif2* (Fig. 3B) and in EGV366, the fcpL was located 640 kb away from it (Fig. 3C). Both fcp were located close to their corresponding midpoint, at less than 1.5% of each replicated DNA segment (Fig. 3B,C, Table S5). These results show that replication forks may progress beyond *dif2* over ~67% of the left replichore of chr2 and over the entire right replichore of chr2. Taken together, these data led us to conclude that no RFT restricts the completion of replication within Ter1 or Ter2.

### Impacts of a premature replication of Ter

**Figure 4:**
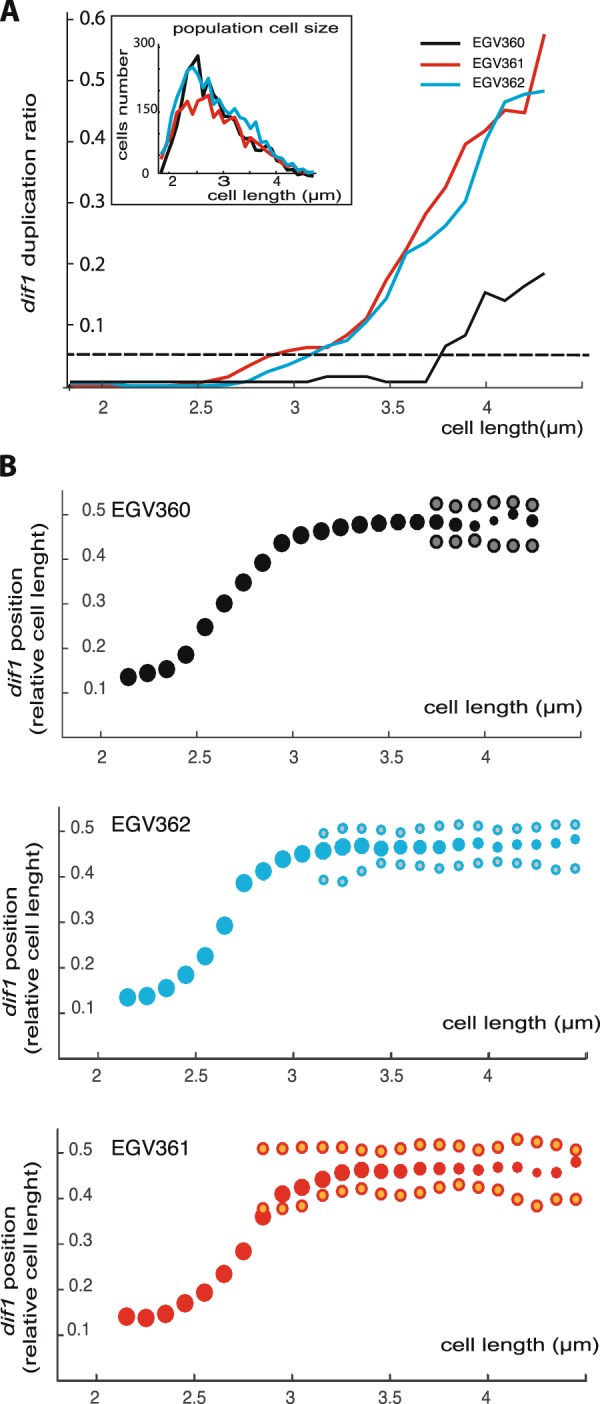
Duplication and choreography of *dif1* locus in WT and ectopic *oriC1*-containing derivatives. (**A**) Proportion of cells with 2 *dif1*-foci according to the cell length (cell size intervals of 0.1 µm). Population cell size is represented in the inset. (**B**) Choreography of *dif1* in EGV360 (top panel), in EGV362 (middle panel) and in EGV361 (bottom panel). The relative positioning of *dif1* along the cell length axis is in function of the cell length (cell size intervals of 0.1 µm); For each size interval, the median positioning of *dif1* among the cells with 1 *dif1*- focus (plain dot) and the pair of median *dif1* positioning among the cells with 2 *dif1*-foci (bi-coloured dot) are indicated (when proportion exceed 5% as shown by the dashed line in (**A**). The size of the dots reflects qualitatively the proportion between the 1- and the 2-*dif1* cells among each size interval. (0: new pole; 1: old pole). For each strain, 2500 cells minimum were analysed.

Because of the presence of *oriR4*, *dif1* is replicated 600 kbp before the fcpL in EGV111. Similarly, because of *oriL3*, *dif1* is replicated about 350 kbp before the fcpR in EGV140. Using strains with fluorescent tags allowing to visualise *dif1* and *dif2* foci, the fraction of cells with duplicated *dif1* and the localisation of *dif1* as a function of cell length were analysed in *oriL3*- and *oriR4*-containing strains (EGV362 and EGV361, respectively) and compared to the corresponding WT strain, EGV360 (Fig. 4). The cell length distribution of the 3 strains were similar (Fig. 4A inset). We observed a higher proportion of cells with two *dif1* foci in the 2.5 to 4.2 µm sizes of strains EGV361 and EGV362 than in the WT strain EGV360 (Figs 4A, S4). In agreement with the marker frequency data, it suggested that *dif1* replication occurred earlier in the cell cycle in EGV361 and EGV362 cells than it did in WT EGV360 cells. More importantly, *dif1* loci still globally behaved like genuine terminus DNA in EGV362 and EGV361 strains: in most of the cases, *dif1* foci only duplicated after the mobilisation of the focus to mid-cell (Fig. 4B,^52^). It is noteworthy that duplicated *dif1* copies remained close to each other, near mid-cell in EGV361 and EGV362 cells, even if duplication occurred an earlier stage of the cell cycle (i.e. in shorter cells) than in EGV360. This likely explains why strains EGV361 and EGV362 exhibited no obvious defect in the coordination of chromosome segregation and cell division. In particular, we observed no anucleated nor filamentous cells.

## Discussion

Our results unambiguously demonstrate that no replication fork trap (RFT) exists in *V. cholerae*. The addition of an extra origin of replication on the chromosome of *V. cholerae* resulted in a modification of the DNA synthesis program such that replication terminated about halfway from the origin(s), irrespective of the chromosomal region, not necessarily around the *dif* site nor within Ter, for both chr1 or chr2.

The absence of RFT on chr2 was already suggested in *V. cholerae*^55^. A strain in which the origin of replication of chr2 is inactivated may propagate, granted that chr2 fuses with chr1. In one such strain, the two chromosomes fused through their *dif* sites sequences. As a consequence, the orientation of the chr2 sequence was inverted with respect to that of its replication when autonomous and any functional replication fork trap would have forbidden the completion of replication, which would have prevented the propagation of the strain. However, it was not possible to exclude that the strain, obtained at low frequency, had acquired a secondary mutation that prevented the conjectural RFT.

*In E. coli*, initiation of replication at ectopic origins may be silenced, depending on their genomic location. When functional, their initiation frequency appears similar to that of the origin on its endogenous context, independently of its position or the length of the origin fragment used (from 449-bp to 5-kbp)^36,50^. We observed in some of our *V. cholerae* MFA that the copy number of the 384-bp ectopic origin (*oriR4* in Fig. 3) is higher than that of *oriC1* at its endogenous locus. This could be explained by the absence of a negative cis-regulation of the initiation of replication on the 384-bp fragment. The absence of the negative regulation may be masked by the perturbation created by the transcription of *rrnE* (*oriL3*) and by the different growth conditions used in our MFA, as judged by the different level of *oriC1*/fcpR ratio between the replicates (*oriR4*: 1.21 and 1.14).

Our results further showed that a fork progressing in the opposite direction of a *rrn* operon transcription was impeded (Figs 2, 3 and S6). Transcription *rrn* increases as a function of the richness of the medium, likely explaining the higher level of replication perturbation when the strain was grown in rich medium. These transcription-replication conflicts may explain the lower level of MF observed at the *oriL3* and *crtS* loci because of local DNA degradation in the *rrnE* region. Consequently, the copy number of chr2, whose replication initiation depends on the duplication of the *crtS* locus located on chr1, was lower in the strain grown in the rich medium than in the strain grown in the medium (Figs 2 and S6). However, no dramatic perturbation was observed when forks progressed on an illegitimate replichore around the Ter regions. Furthermore, our results indicated that replication completion at Ter was not required to ensure the proper coordination between chromosome segregation and cell division (Figs 2, 4).

The MFA showed that replication terminates very accurately near *dif* in cultures of WT *V. cholerae* (Fig. 2). In contrast, the deviation of the fork convergence points (fcp) from the midpoints between two origins (mp) and the increased zone of termination (S95 value) indicated that the robustness of the process was significantly reduced in strains in which an additional origin of replication was integrated elsewhere on the chromosome (Table S4 and S5). It suggests that the progression of replication forks is perturbed on illegitimate replichores. In bacterial genomes, strongly expressed genes such as those contained in ribosomal operons (*rrn*) are usually transcribed in the same direction as the progression of the replication forks. Hence, the difference in terms of precision of replication completion between the WT and the two mutant strains could reflect the deleterious consequences of opposite transcription and replication orientations. In EGV140, containing an ectopic *oriC1* close to *rrnE*, the distance between fcpL and mpL was probably increased linked to the *rrnE* perturbation; in EGV111, the probability to terminate far from fcpR or fcpL (S95 parameter) was probably increased by perturbations on each side of fcpR or fcpL. Head-to-head collisions between transcription bubbles and replication forks were shown to lead frequently to replication fork inactivation in fast growth conditions in *E. coli* to the point that *recA* became essential in strains in which the orientation of *rrn* operons were inverted^37^. Similarly, doubling time was reduced by a combination of ectopic origins that would rebalance the replichore size^36^. These replication hazards had also an effect on growth rate in *V. cholerae*, although to a lesser extent (Table S2).

We initiated this work with the idea that in *E. coli* RFT might participate in the positioning of Ter at mid-cell at the time of cell division by forcing termination in this region^56,57^. However, our data demonstrate that whatever the replication timing, *dif1* sister loci remain colocalised until their positioning at mid-cell at the time of division in *V. cholerae* (Fig. 4A). Hence, we envision that the spatial arrangement of the chromosome during the cell cycle and the associated coordination between chromosome segregation with cell division could be the rationale behind the presence of an RFT system in some organisms and not in others. In contrast to *V. cholerae*, the chromosome of *E. coli* follows a Left-Right transversal arrangement, implying that merely the region where replication is completed, and therefore not necessarily Ter, is located at mid-cell. Indeed, modifications of the RFT zone or perturbations of one of the two replication forks progression have important consequences on the spatial positioning of the Ter region in *E. coli*^58,59^. Assuming that the localization of Ter is critical for the formation of the septal ring at mid-cell, the Left-Right arrangement of the chromosome would benefit from the assistance of an RFT to force the localisation of Ter at mid-cell when replication is completed. Given that only a few species have a Left-Right chromosome arrangement, it is likely that RFT acquisition followed the loss of the *ori*-*ter* arrangement to circumvent problems associated with Ter mispositioning.

Only a few bacteria encode an RTF system. We observed that the *ter*/Tus system is carried by plasmids that proliferate in γ-proteobacteria and we proposed that, in the course of evolution, *tus* was directly or indirectly domesticated only thrice and relatively recently within this bacterial class. The absence of such a system in *Plesiomonas shigelloides*, an atypical enterobacteria that naturally lives in brackish waters and that diverged early during the evolution of the Enterobacteriales, suggests that the domestication occurred simultaneously with or in response to the switch to another ecological niche in that order. Finally, the absence of RFT in *V. cholerae*, and in most bacteria, strongly suggests that the domestication of the *ter*/Tus system was not achieved at the expenses of an ancestral system, but rather as a gain of function, possibly to ensure an improved proliferation rate of the cells.

## Methods

### Plasmids and strains

Bacterial strains and plasmids used in this study are listed in Table S1. All *V. cholerae* mutants were constructed by integration-excision or natural transformation^52^. To this end, derivatives of the El Tor *V. cholerae* N16961 and MCH1^24^ were rendered competent by the insertion of *hapR* by specific transposition^60^. Description of strain construction is included in Sup MM.

### Genomic DNA bank preparation for MFA

Cells were grown in M9 minimal medium supplemented with 0.4% fructose or in LB rich medium to exponential phase (0.05 and 0.2 OD at 650 nm in M9 and LB, respectively). Chromosomal DNA was extracted using the Sigma GenElute® bacterial genomic DNA kit to generate a genomic library according to Illumina’s protocol. The libraries and the sequencing were performed by the High-throughput Sequencing facility of the I2BC (http://www.i2bc.paris-saclay.fr/spip.php?article399&lang=en, CNRS, Gif-sur-Yvette, France). Genomic DNA libraries were made with the ‘Nextera DNA library preparation kit’ (Illumina) following the manufacturer’s recommendations. Library quality was assessed on an Agilent Bioanalyzer 2100, using an Agilent High Sensitivity DNA Kit (Agilent technologies). Libraries were pooled in equimolar proportions. 75 bp single reads were generated on an Illumina MiSeq instrument, using a MiSeq Reagent kit V2 (500 cycles) (Illumina), with an expected depth of 217X.

### Marker frequency analysis

An in-lab written MATLAB-based script (available on demand) was used to perform marker frequency analysis. The determination of the termination parameters (fork convergence point (fcp) and S95) are described in Supp. Methods. The MFA data have been submitted to the ArrayExpress repository. The access number for these data is E-MTAB-7193.

### Fluorescence microscopy

Cells were grown in M9 minimal medium supplemented with 0.4% fructose to exponential phase and spread on a 1% (wt/vol) agarose pad for analysis. Cell images were acquired using a DM6000-B (Leica) microscope with MetaMorph software (Version 7.8.8.0, Molecular Devices). Cell outlines and spot positions were determined using Microbetracker^61^. More than 2500 cells were analysed in each experiment. Orientation of the cells containing only one *dif1* focus was arbitrary: the pole closer to *dif1* was called pole 0.

## Supplementary information

Supplementary information
