# Supplementary material for: Replication termination without a replication fork trap
Source: Sci Rep. 2019 Jun 5;9:8315. doi: 10.1038/s41598-019-43795-2 (PMC6549158; doi:10.1038/s41598-019-43795-2)
Supplement: Supplementary file 1 — Supplementary information [file 41598_2019_43795_MOESM1_ESM.docx]

**Supplementary information**

**Replication termination without a replication fork trap**

Elisa Galli^1*^, Jean-Luc Ferat^1,2*^, Jean-Michel Desfontaines^1*^, Marie-Eve Val^3,4^, Ole Skovgaard^5^, François-Xavier Barre^1,#^ and Christophe Possoz^1,#^

**Supp. Figures (Fig. S1 to Fig. S7)** p. 2……………..p.8

**Supp. Tables (Table S1 to Table S5)** p. 9……………..p.11

**Supp. Materials and Methods** p.12 ……………p. 19

**References** p. 20


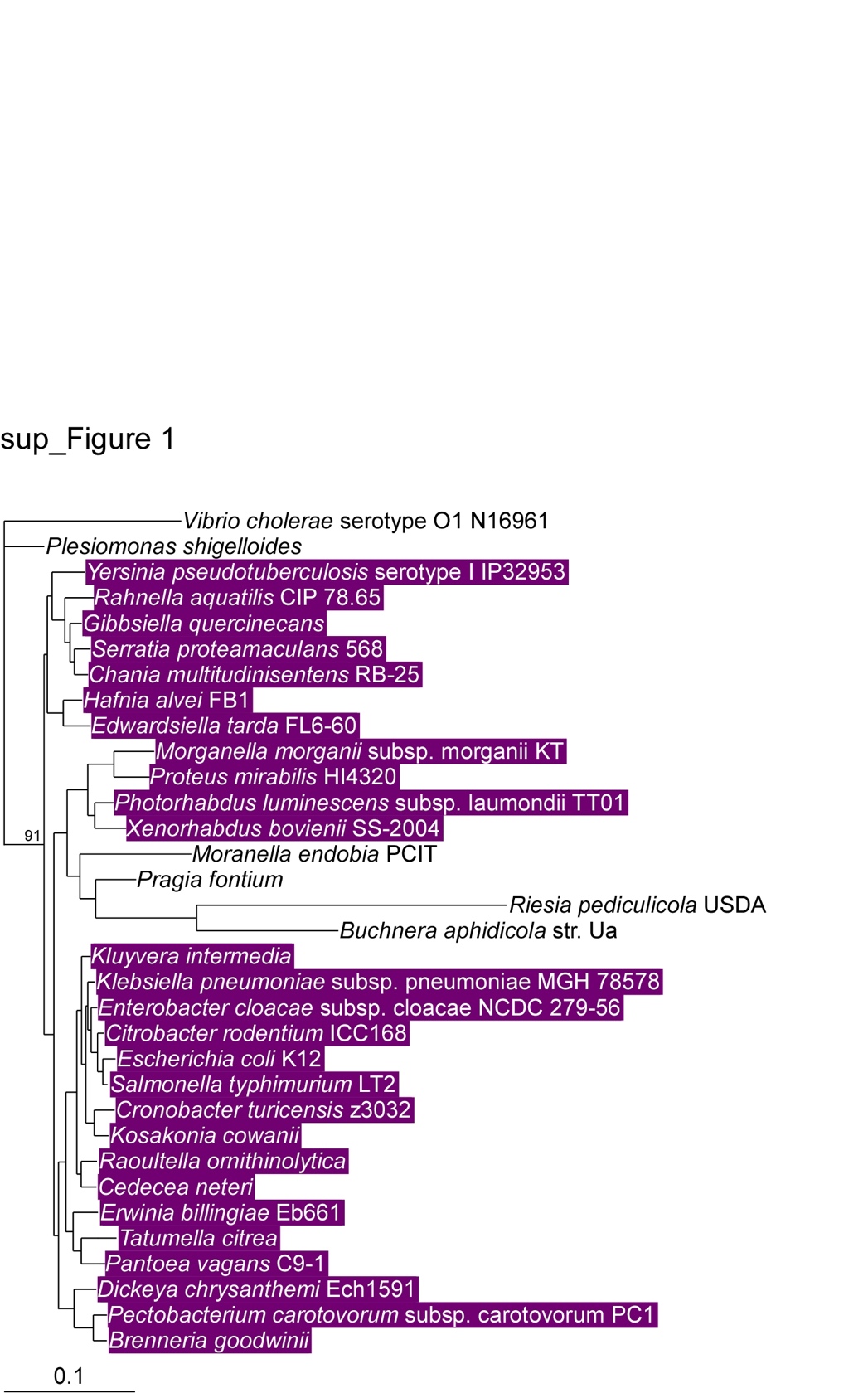


Fig. S1: Prospective domestication of *tus* in enterobacteriales. On the phylogenic tree (based on DnaABEX proteins) of the enterobacteriales using *V. cholerae* as an outgroup, the *tus*-containing species are highlighted in purple (presence of a resident PF05472). *tus* is not present in *Plesiomonas shigelloides* but in all the other enterobacteriales (except for the 4 endosymbionts, not considered in the phylogenetic analysis). The arrow indicates the probable position of *tus* domestication indicated on the figure (bootstrap score of this branch is significant >75). Scale bar represents 0.1 substitution per site.


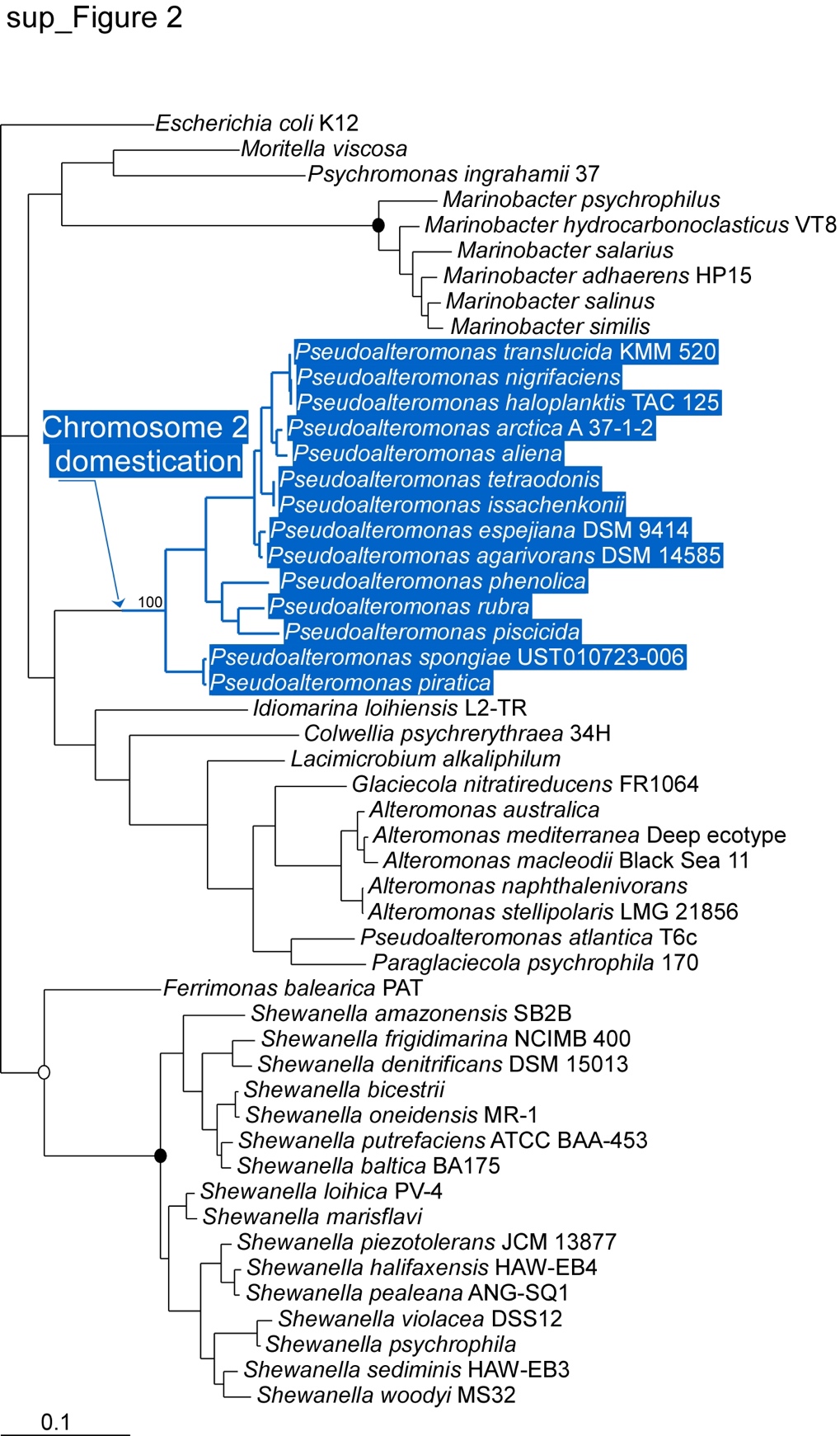


Fig. S2: Prospective domestication of *tus* in A. On the phylogenic tree of the Alteromonadales (based on DnaABEX proteins) using *E. coli* as outgroup, the *t*us-containing species are highlighted in blue. *tus* is present is a monophyletic branch within the Alteromonadales. The prospective domestication was concomitant with that of a second chromosome in the genus *Pseudoalteromonas*. The position of domestication is indicated by the arrow on the figure and a number corresponding to the bootstrap score of this branch. Scale bar represents 0.1 substitution per site.


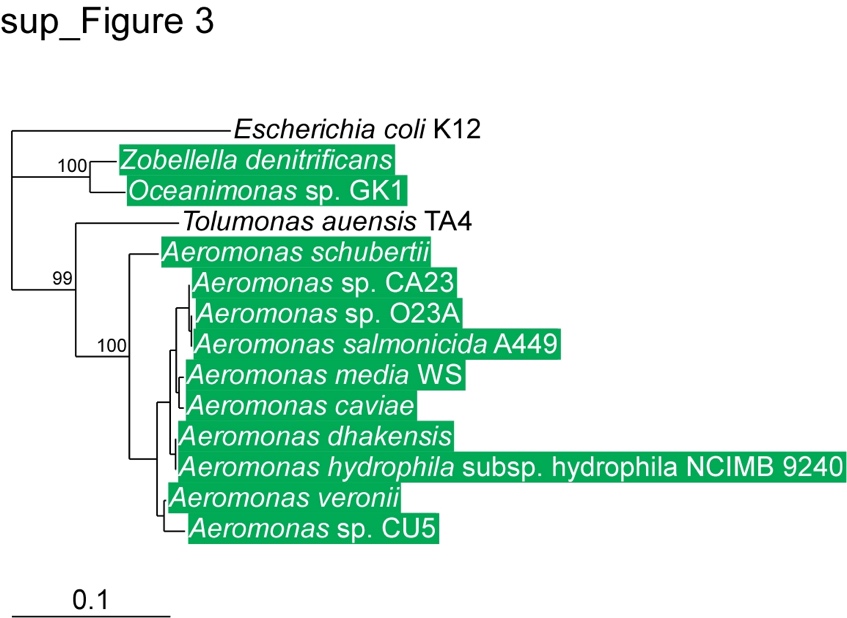


Fig. S3: Prospective domestication of *tus* in Aeromonadales. On the phylogenic tree of the Aeromonadales (based on DnaABEX proteins) using *E. coli* as outgroup, the *tus*-containing species are highlighted in green. *tus* is present in all the species (except *Tolumonas aurensis*) suggesting that *tus* domestication occurred with the emergence of the Aeromonadales branch. Scale bar represents 0.1 substitution per site. Significant bootstrap scores (>75) are indicated.

2 *ter1* / 1 *ter2*

1 *ter1* / 2 *ter2*

2 *ter1* / 2 *ter2*

**Fig. S4: Histograms of the proportion of the different cell types (based on the number of *ter1* and *ter2* foci)**

More than 2500 cells analyzed for each strains. *ter1* is a locus at 1kb from *dif1* site and *ter2* at 1kb from *dif2*. In the WT context (EGV360), the proportion of cells with two *ter2*  (3+8=11%) is much higher than the proportion with two *ter1* (3+1=4%) confirming that *ter2* duplication happened before *ter1* duplication ^1^.

In presence of the ectopic *oriC1* at position L3 (EGV362), the proportion of *ter2*duplication is even larger (11+6=17%), as expected by the earlier timing of chromosome 2 replication initiation due to *crtS* earlier replication. In addition, the proportion of cells with *ter1* duplication is also larger (6+3=9%).

In presence of the ectopic *oriC1* at position R4 (EGV361), the proportion of *ter2* duplication is not very different from that measured in the WT context (6+6=12% as compared to 11%), as expected by the unmodified timing of replication of *crtS* in this strain. However, *ter1* is duplicated much earlier.

All these data confirm that the modification of *crtS* replication timing (from MFA) leading to modification of *ter2* replication timing, modifies *ter2* duplication timing in a similar manner.

**Table S1**. List of bacterial strains and plasmids

| **Strains** |  |  |  |
| --- | --- | --- | --- |
| **Name** | **Relevant genotype or features** | **Reference** | |
| EPV50 | N16961 *ChapR* Δ*lacZ* gm^R^ | ^2^ | |
| EGV140 | N16961 *ChapR* Δ*lacZ oriC1* @ L3 (0.774Mbp) gm^R^ | This study | |
| EGV111 | N16961 *ChapR* Δ*lacZ oriC1* @ R4 (1,898Mbp) gm^R^ | This study | |
| MCH1 | Integration of chromosome 2 deleted of oriC2 in place of the dif1 site in chromosome 1 | ^2^ | |
| EGV369 | MCH1 *ChapR oriC1* @ L3 (0.774Mbp) gm^R^ | This study | |
| EGV366 | MCH1 *ChapR oriC1* @ R4 (1,898Mbp) gm^R^ | This study | |
| EGV360# | N16961 ChapR ΔlacZ (lacI-RFP-YGFP-parBT1) Δdif1::tet-parST1-tet-dif1 (terI) + LacOarray-Kan inserted on chrII at position terII : 0,5 Mb (497861 bp) | This study | |
| EGV362 | EGV360 *oriC1* @ L3 (0.774Mbp) | This study | |
| EGV361 | EGV360 *oriC1* @ R4 (1,898Mbp) | This study | |
| **Plasmids** |  |  | |
| **Name** | **Relevant genotype or features** | **Reference** | |
| pPOS228 | pUC derivative ; *oriC1*-zeoR between homology zones around L3I ; AmpR, zeoR | ^2^ | |
| pAD39 | *ParST1*-frt-CmlR-frt between TetR homologies. | ^2^ | |
| pEP70 | oriR6K; CmlR; SacB; YGFP-ParBT1 and LacI-RFT between Up and Dwstream regions of *V.cholerae* LacZ | ^1^ | |
| pAD20 | LacO array + KanR between TetR homologies; KanR; CmlR; SacB; ori R6K | ^2^ | |
| pNB1* | pUC vector ; oriC1-CmR between TetR homology zones; AmpR, CmR | This study | |

* pNB1 was constructed by cloning of the oriC1 fragment DraIII/PstI from pPOS228 into pAD39 restricted by AlwNI/ PstI.

# EGV360 was constructed using strains containing Tet homologies inserted near *dif1* and *dif2*, lacO and parS PMT1 sites were added using pAD20 and pAD39 respectively . pEP70 was used to express YGFP-ParBT1 and LacI RFT from the chromosome

**Table S2. Growth rate parameters of two-chromosome *V. choleare* strains.**

| Strain/medium | GT +/- SD (mins) | ori1 | S1a | fcp1 or 1R | S1b | oriL3/R4 | S1b' | fcp1L | S1a' |
| --- | --- | --- | --- | --- | --- | --- | --- | --- | --- |
| EPV50 MM | 50 +/- 4 | 1.54 | -4,3E-07 | 1.00 | n/a | - | n/a | - | 4,3E-07 |
| EGV140 MM | 55+/- 3 | 1.54 | -5,3E-07 | 1.00 | -3,0E-07 | 1.52/1.42 | 6,0E-07 | 1.36 | 5,3E-07 |
| EGV111 MM | 55+/- 5 | 1.32 | -4,7E-07 | 1.09 | -4,7E-07 | 1.32 | 4,4E-07 | 1.00 | 4,6E-07 |
|  |  |  |  |  |  |  |  |  |  |
|  | ori2 | S2 | fcp2 | S2' | crtS |  |  |  |  |
| EPV50 MM | 1.22 | -5,6E-07 | 1,0E+00 | 5,0E-07 | 1.26 |  |  |  |  |
| EGV140 MM | 1.40 | -6,0E-07 | 1.13 | 5,2E-07 | 1.49 |  |  |  |  |
| EGV111 MM | 1.03 | -3,7E-07 | 0.91 | 3,0E-07 | 1.06 |  |  |  |  |
|  |  |  |  |  |  |  |  |  |  |
| Strain/medium | GT +/- SD (mins) | ori1 | S1a | fcp1 or 1R | S1b | oriL3/R4 | S1b' | fcp1L | S1a' |
| EPV50 LB | 22 +/- 1 | 2.78 | -1,18E-06 | 1.00 |  | - |  | - | 1,23E-06 |
| EGV140 LB | 25+/- 1 | 3.08 | -1,27E-06 | 1.00 | 1,20E-06 | 2.47/2.07 | -1,88E-07 | 2.05 | 1,5E-06 |
| EGV111 LB | 25+/- 1 | 1.87 | -9,80E-07 | 1.21 | 8,04E-07 | 1.6 | -8,13E-07 | 1.00 | 9,23E-07 |
|  |  |  |  |  |  |  |  |  |  |
|  | ori2 | S2 | fcp2 | S2' | crtS |  |  |  |  |
| EPV50 LB | 1.45 | -1,18E-06 | 0.96 | 1,27E-06 | 1.71 |  |  |  |  |
| EGV140 LB | 1.76 | -1,33E-06 | 1.08 | 1,32E-06 | 2.35 |  |  |  |  |
| EGV111 LB | 1.03 | -1,32E-06 | 0.63 | 1,28E-06 | 1.19 |  |  |  |  |

The columns starting from the left indicate : **1**: the strain name and the medium used for the culture (MM: Minimal medium; LB: Luria Broth; **2**: The Generation Time (GT) with standart deviation (SD) in mins; **3, 5, 7, 9, 11, 13, 15**: the marker frequencies (MF) of *ori1*, fcp1 or fcp1R, *oriL3* or *oriR4*, fcp1L, *ori2*, fcp2, *crtS*, respectively. **4, 6, 8, 10, 12, 14**: Slopes of each replication arms (from MF in log2), called S1a and S1a’ for slopes emerging from *ori1* (blue columns), S1b and S1b’ emerging from *oriL3/R4* (green columns) and S2 and S2’ emerging from *ori2* (red column). All MF are normalised by the value of the lowest fcp of the chromosome 1 (grey box). In the case of oriL3, the table indicates the MF values obtained for the right and left replication arms, which were markedly different leading to different slopes S1b and S1b’(yellow boxes).

**Table S3. Growth rate parameters of the one-chromosome strains used.**

| Strain/medium | GT+/- SD (mins) | ori1 | S1a | fcp or fcpR | S1b | oriL3-R4 | S1b' | fcpL | S1a' |
| --- | --- | --- | --- | --- | --- | --- | --- | --- | --- |
| MCH1 MM | 66 +/- 1 | 1.43 | -2,53E-07 | 1.00 | - | - | - | - | 2,57E-07 |
| EGV369 MM | 64 +/- 1 | 1.34 | -2,42E-07 | 1.00 | 2,70E-07 | 1.37/1.30 | -8,80E-09 | 1.30 | 1,69E-07 |
| EGV366 MM | 71 +/- 6 | 1.28 | -1,91E-07 | 1.20 | 4,13E-07 | 1.43 | -3,58E-07 | 1.00 | 2,50E-07 |

Legend as in Table S2.

**Table S4. Termination Parameters in two-chromosome *V. choleare* strains.**

|  | R(fcp1 or fcp1R) | | R(fcp1L) | | R(fcp2) | |
| --- | --- | --- | --- | --- | --- | --- |
| Strain/medium | mp-fcp | S95 | mp-fcp | S95 | mp-fcp | S95 |
| EPV50 MM | -0,1% | 0,1% | - | - | 0,04% | 0,8% |
| EGV140 MM | 0,6% | 27% | -1,6% | 20% | -1,2% | 0,4% |
| EGV111 MM | -0,2% | 7,4% | -0,8% | 22% | -0,5% | 26% |
| EPV50 LB | 0,5% | 23,3% | - | - | -0,2% | 37,3% |
| EGV140 LB | 3,1% | 69,1% | -11,4% | 64,4% | -0,2% | 38,8% |
| EGV111 LB | 3,0% | 99,6% | -4,1% | 154,4% | 0,4% | 108,9% |

The columns starting from the left indicate : 1) the strain and the medium used for the culture (MM: Minimal medium; LB: Luria Broth); 2) to 4) the replicons (R) corresponding to the different fork convergence points: R(fcp1 or fcp1R), R(fcp1L) andR( fcp2). For each replicon is indicated: mp-fcp and σ. mp-fcp is the replicon proportion (in %) between the replicon midpoint (mp) and the fcp and S95 is the replicon proportion (in %) of the region centred on fcp containing 95% of the termination events. S95 equals 4σ with σ being the sigma of a gaussian representing the proportion of cells in which termination occurred various distances from fcp (See **Supp. Materials and Methods** for explanations).

**Table S5. Termination Parameters in one-chromosome *V. choleare* strains.**

|  | fcp or fcpR | | fcpL | |
| --- | --- | --- | --- | --- |
| Strain/medium | mp-fcp | S95 | mp-fcp | S95 |
| MCH1 MM | -0,3% | 0,10% | - | - |
| EGV369 MM | 0,9% | 8,86% | * | * |
| EGV366 MM | -1,4% | 5,37% | 1,0% | 22,80% |

Legend as in Table S4.

* Perturbation of MF data preventing our fitting methods

**Supp Materials and Methods**

**Strain Construction**

-EGV140, EGV111, EGV360, EGV366, EGV361 and EGV362: The ectopic origin *oriC1* used in this study correspond to 384bp of chr1 from 5’ CCTATTCCATGCAGAGCGGC 3’ to 5’ TCCTGTTTTTTCGATCAAGG 3. This fragment was cloned between two fragments of about 1kb corresponding to the position of the desired integration by natural transformation either generating pPOS228 plasmid for L3 position or PNB1 plasmid to target tetR homologies integrated at R4 position by transposition.

-The elements used in EGV360 to visualize terI and terII were already used in^2^.

**Phylogenetic analysis of *tus*genes**

Tus proteins were first identified through their HMM signature, recorded as the Pfam domain PF05472. The distribution of *tus* in enterobacteriales is erratic, suggesting that some tus genes, at least, are associated with mobile elements. In this study, we are interested in *tus* genes transmitted vertically, i.e., in *tus* that are “resident”. We established residency when 1) the distribution of Tus was monophyletic and congruent (meaning consistant) with the phylogenetic tree of the species in which *tus* is found (We used a concatenation of the DnaABEX protein to establish the phylogeny of the species) and 2) the *tus* genomic context was conserved within the monophyletic group. The consistency between the two criteria was evaluated qualitatively. In the phylogenetic tree, the “time” of domestication is rooted to the branch encompassing the entire group of resident *tus* deriving from a common ancestor.

**Marker Frequency Analysis**

**A.** Single reads were generated on an Illumina MiSeq instrument, using a MiSeq Reagent kit V2 (500 cycles) (Illumina). In the order of 2x10^7^ reads were recovered for each sample. We used the BWA software to map them on the genome of the cognate *V. cholerae* strain, which was built *in silico* with SnapGene. uniquely mapping sequence read numbers were normalised by by the total number of unique reads obtained for the sample.

Enrichment of uniquely mapping sequence reads were calculated over 1kb and 200kb sliding windows. Local 1kb-window values deviating by more than 15% from the local 200kb-window values were discarded from the analysis. For the most part, they corresponded to regions of low GC content that were associated with mobile elements, such as the Super Integron on chr2 and the VPI-1, VPI-2, VSP-1 and VSP-2 Vibrio pathogenicity islands on chr1 (see Figure S11 as an example). To further avoid any motif specific sequencing bias, local 1kb-window values obtained with the genomic DNA extracted from exponentially growing cells were normalised with the 1kb-window values obtained with the genomic DNA extracted from the cells in stationary phase.

**B.** Assuming a constant speed of replication, the number of copies per genome equivalent (the marker frequency, $MF$) of a locus in an exponentially growing asynchronous population is given by is given by $MF\left( x \right)=k2^{ax}$, where $x$ is the fractional distance of the locus from the origin and terminus of replication of the replication arm on which it is located, and $k$ and $a$ are two constant ^3^. The average fork convergence point of a chromosome region comprised between two origins (o and o’) was determined as the position of replication termination, $t$, that would minimise a gaussian fitting the inverse bell shape curve given by the quadratic error between the $log2$of the experimentally determined $MF$, $Y$, and the corresponding linear regression of the $Y$ values on each replication arm, $y$:

$e(t)=\sum_{p=0}^{p=0'} \frac{{(Y-y)}^{2}}{N}$, where $N$ is the number of data points and $y\left( x \right)=ax+n$ with $x=\frac{p-o}{t-o}$ if $o<p<t$and $y\left( x \right)=a'x+b'$ with $x=\frac{p-o'}{t-o'}$ if $t<p<o'$ (Figure S***).

Illustration of the method used to determine the average fork convergence point. Top panels: linear regression (red segments) of the $log2$ of the MF data (blue points) on a chromosomal region comprised between two origins. Top left panel: the termination point was set at ¼ from the left origin.Top right panbel: the termination point was set at ½ the distance between the two origins (right). Bottom panel: quadratic error between the obtained theoretic and experimental values (blue points). The gaussian best fitting the error curve is drawn in red.

**C.** Estimation of the frequency at which fork convergence occurred at various distances from fcp in the analysed cell populations (σ). In the case of EPV50 and MCH1, the $log2$of the MF values, $Y$, around fcp1 and fcp2 was well explained by the linear regression segments derived from the Cooper-Helmstetter model. In the strains harbouring extra-numerous ectopic origins, $Y$ seemed less sharp. It suggested that the position of fork convergence could be less precise, i.e. that in some cells of the analysed population fork convergence occurred at a different point than the average fcp calculated in B. We considered that the frequency at which fork convergence occurred at a given distance from fcp could be represented by a gaussian centred on fcp, $g_{fcp,\sigma}\left( t \right).$ The larger the σ of this gaussian, the higher would be the fraction of the cells in which fork convergence deviated from fcp in the analysed population. The theoretic $log2$ of the MF values, $y$, would then be:

$y_{fcp,\sigma}\left( x \right)=\sum_{t=0}^{t=o'} y_{t}\left( x \right).g_{fcp,\sigma}(t)$, where $y_{t}\left( x \right)=\left( Y_{o}-Y_{fcp} \right).x+Y_{fcp}$ with $x=\frac{p-o}{t-0}$ if $o<p<t$, and $y_{t}\left( x \right)=\left( Y_{o'}-Y_{fcp} \right).x+Y_{fcp}$ with $x=\frac{0^{'}-p}{0^{'}-t}$ if $t<p<o'$

σ was then chosen as the value that minimised the quadratic error between $Y$ and $y_{fcp}$:

$e(\sigma)=\sum_{p=0}^{p=0'} \frac{{(Y-y_{fcp,\sigma})}^{2}}{N}$

Illustration of the method used to take into consideration the frequency of fork convergence around the average fcp. Left panel: σ= 1; Right panel: σ= 10. Blue line: gaussian probability of fork convergence at each lous, $g_{fcp,\sigma}\left( t \right).$ Black lines: Cooper-Helmstetter theoretic $log2$ of the MF values for various positions, t, of fork convergence, $y_{t}\left( x \right)$. The thickness of the lines represent their relative contribution to marker frequencies. Red line: $y_{fcp,\sigma}\left( x \right)=\sum_{t=0}^{t=o'} y_{t}\left( x \right).g_{fcp,\sigma}(t)$.

**Validation of our MFA method to determine fcp and S95**

In order to validate our method to define the fcp (termination point), that remains formelly unknown, we decided to use it to determine the origin point.

We used it on two replicate of our WT strain grown in two independent culture: FX85 and FX296 each normalised by its stationary phase FX86 and FX294 respectively, using a sliding window of 1kbp.

EPV50: M9 Exp/Stat): FX85 /FX86 (replicate 1) – origin positioned at 0 and 1, to determine Ter


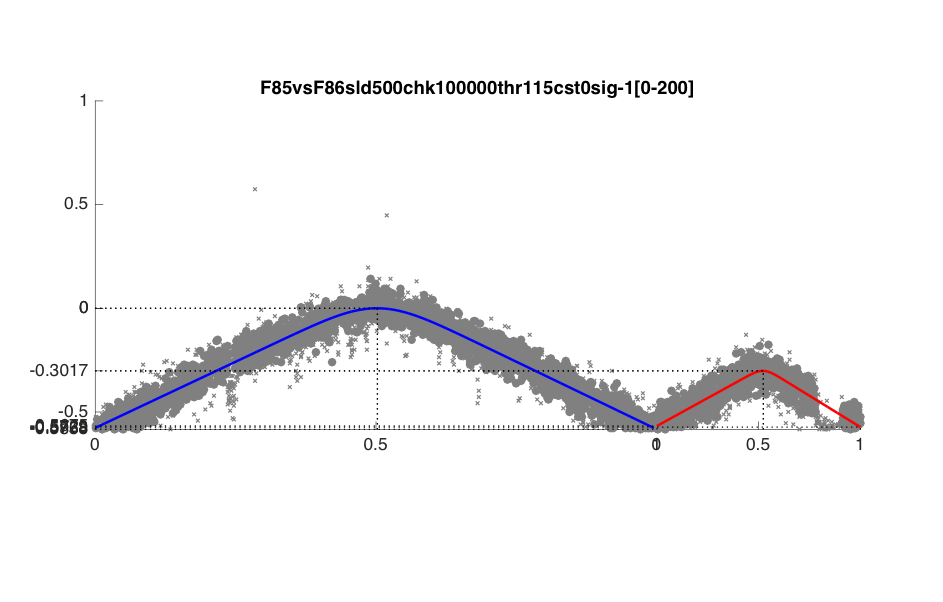


EPV50: M9 Exp/Stat): FX296/FX294 (replicate 2)


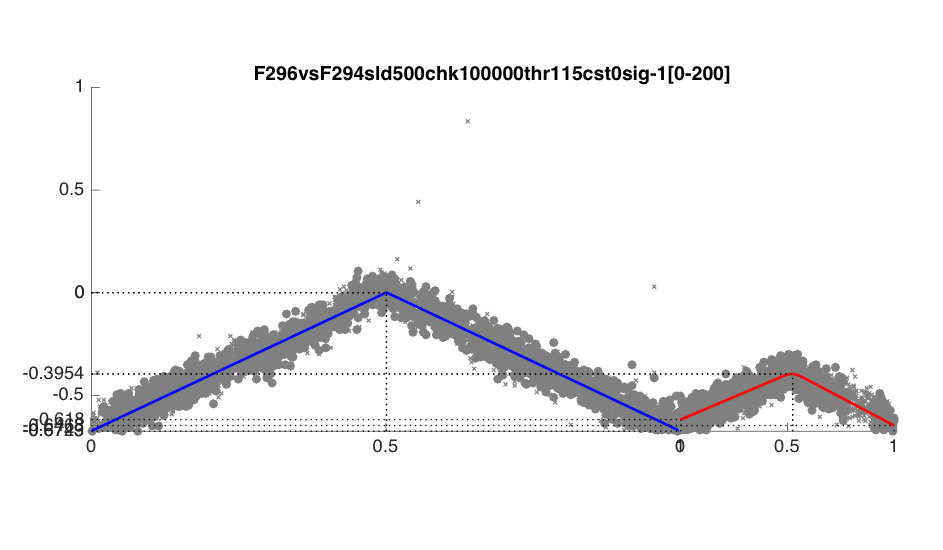


We also used different size of sliding windows (0.5 kbp, 1 kbp and 10 kbp) to evaluate the impact on the origin point and its S95.

| Replicate 1:FX85 | EPV50 |  |  |
| --- | --- | --- | --- |
| Sld size (kb) | 1 | 0.5 | 10 |
| Ori1 | 0,25% | 0,37% | 0,038% |
| S95 | 24,7% | 13,5% | 25,7% |
| Ori2 | 2,28% | 2,42% | 1,30% |
| S95 | 14,5% | 10,3% | 7,5% |
| Replicate 2: FX296 |  |  |  |
| Ori1 | 0,08% | 0,12% | 0,38% |
| S95 | 0,7% | 0,4% | 1,4% |
| Ori2 | 2,5% | 2,7% | 0,36% |
| S95 | 9,3% | 10,6% | 3,7% |

**Ori positions were indicated in % of deviation to the real position in proportion to the size of the replicon concerned. Similarly, S95 was indicated in proportion of the replicon concerned.**

First, we can observe that the ori position and S95 parameter are robust in function of the sliding window. Thus, we decided to use a 1-kb window in the rest of the analysis.

We observe that in both replicate the *ori1* was determined with less than 0.5% of deviation from the origin positions. We can see from the global profile that the first replicate present a “flatter” *ori1* peak which is observe with the higher S95 (2,5%) compared to the second replicate (0,1%). The first replicate might not have a perfect steady state growth. Thus, we demonstrate that ori1 can be precisely positioned,with a good precision parameter (S95) when using good growth conditions. Regarding *ori2*, it seems that the smaller size and/or the presence of the superintegron (a large region presenting uncharacterised biases during the sequencing process) provide less robust data: *ori2* is positioned with more than 2% of deviation from the normal position. It indicates that data concerning the chromosome 2 termination might be less robust.

Then, we applied our MFA method on the termination of the two replicate from our WT strain and applied different sizes of sliding window to confirm that it will not affect the results.

EPV50 (M9 Exp/Stat): FX85 /FX86 (replicate 1) – origin positioned at 0 and 1, to determine Ter


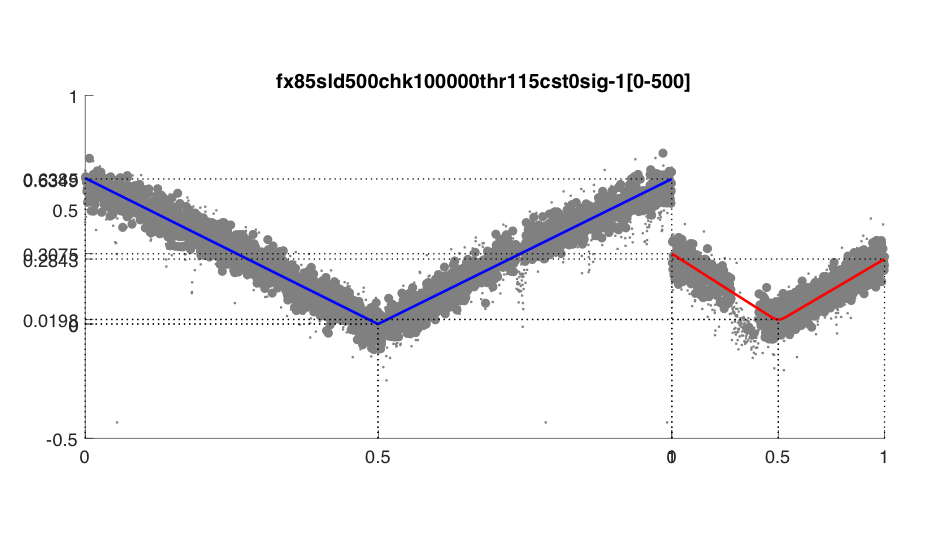


EPV50 (M9 Exp/Stat): FX296/FX294 (replicate 2)


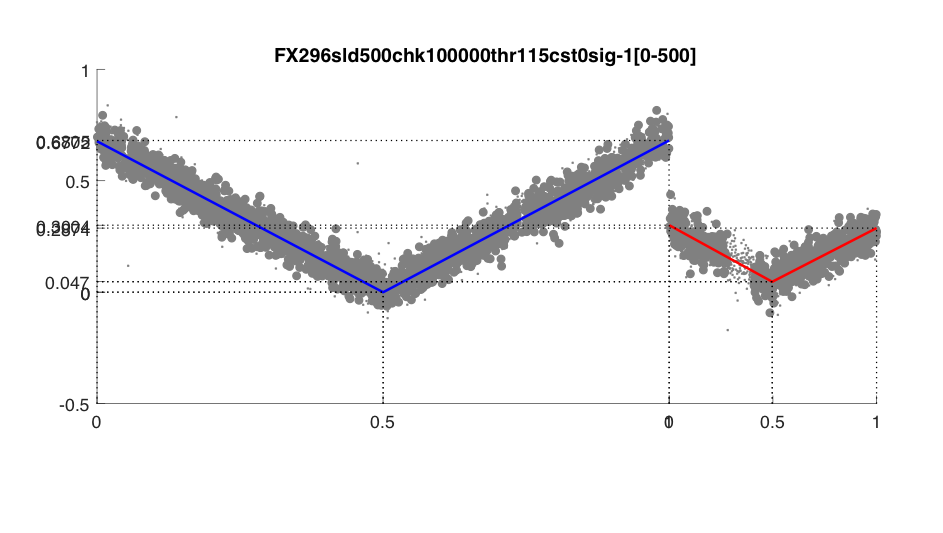


| Replicate 1:FX85 |  | EPV50 |  |  |
| --- | --- | --- | --- | --- |
| Sld size (kb) | 10 | 1 | 0.5 |  |
| Ter1 | -0,30% | -0,09% | -0,30% |  |
| S95 | 1,4% | 0,14% | 0,07% |  |
| Ter2 | -1,50% | 0,04% | 0,23% |  |
| sig2 | 22,4% | 7,83% | 0,19% |  |
| Replicate 2: FX296 |  |  |  |  |
| Ter1 | -0,30% | 0,02% | -0,04% |  |
| sig1 | 13,5% | 0,14% | 0,39% |  |
| Ter2 | 0,36% | -0,43% | -1,36% |  |
| sig2 | 3,7% | 0,37% | 0,19% |  |

As expected the results are not dramatically changed by changing the size of the sliding window and it confirms our choice to use 1kb as sliding window four our MF analysis. The positioning of Ter1 deviated from the midpoint of the chr1 by less than 0.1% with a very small S95 (<0.1%) and Ter2 did not deviate a lot from the midpoint.

Finally, we applied our MFA method on our all the strains of this manuscript (see main text).

We performed replicates for each strains and compared them. As shown below, the reproducibility of the results was satisfactory.

EGV140 (M9 Expo/stat) : FX288/ FX289 (replicate 1)


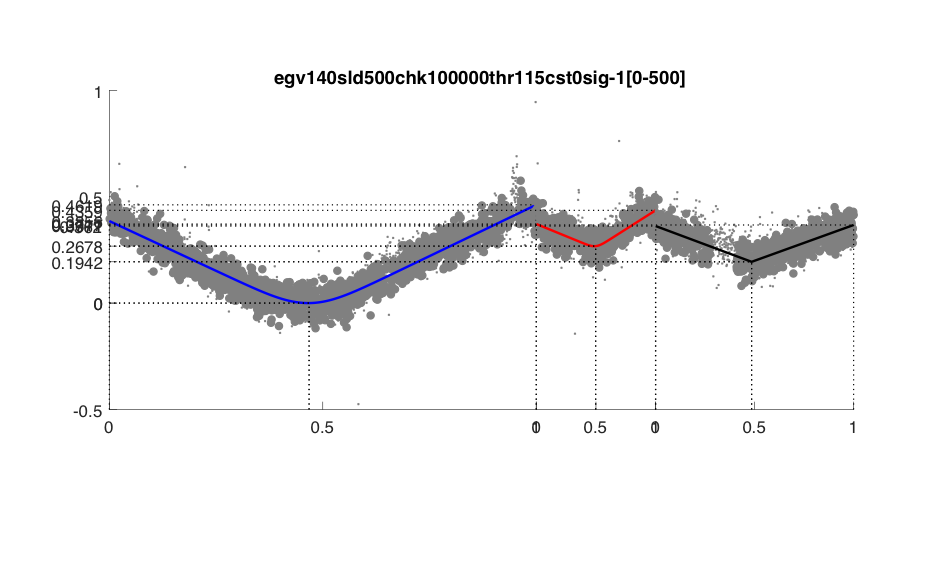


EGV140: (M9 Exp/Stat) FX316/FX294 (replicate 2)


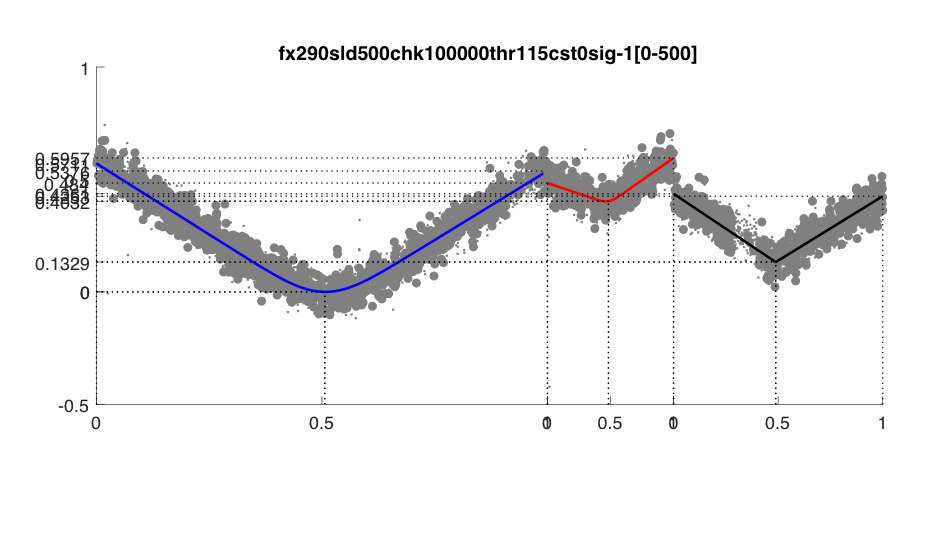


EGV111 (M9 Expo/Stat) : FX290) /FX291 (replicate 1)


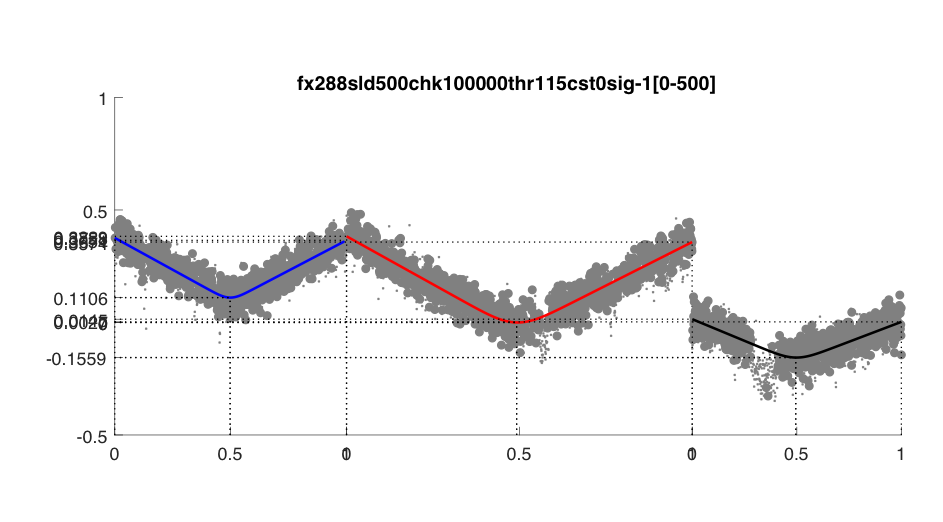


EGV111: (M9 Expo/Stat) : FX315 /FX294 (replicate 2)


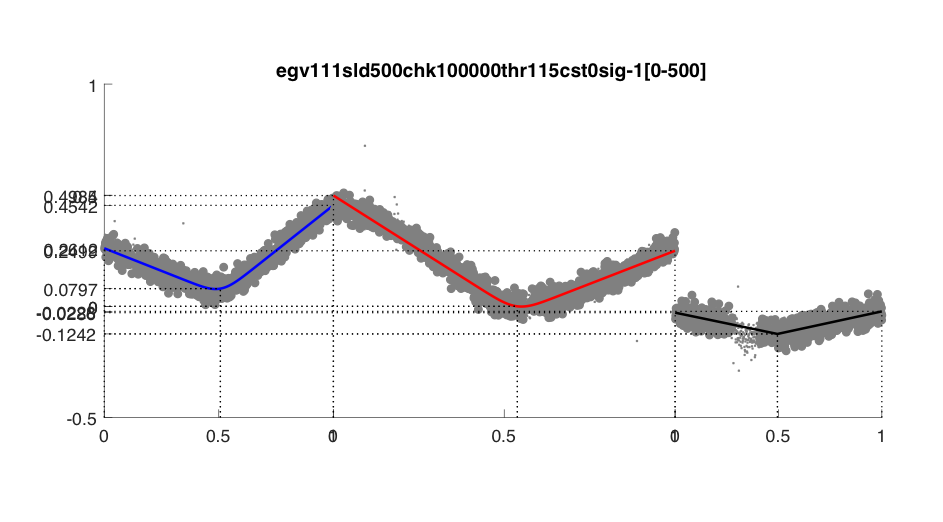


|  |  | EGV111 | | EGV140 | |
| --- | --- | --- | --- | --- | --- |
| Sld size (kb) | | 1 | 0.5 | 1 | 0.5 |
| Ter1a | replicate 1 | -0,2% | 0,35% | 0,6% | 0,8% |
| S95 |  | 7,4% | 0,5% | 27,1% | 27,9% |
| Ter1b |  | -0,8% | -0,8% | -1,6% | 0,1% |
| S95 |  | 22,3% | 25,3% | 19,8% | 17,0% |
| Ter2 |  | -0,5% | -0,05% | -1,2% | -0,8% |
| S95 |  | 26,5% | 22,2% | 0,4% | 10,4% |
|  |  |  |  |  |  |
| Ter1a | replicate 2 | 0,7% | 0,6% | -3,2% | -2,8% |
| S95 |  | 21,2% | 21,7% | 26,8% | 25,6% |
| Ter1b |  | 3,8% | 2,8% | -0,2% | 1,0% |
| S95 |  | 16,3% | 17,4% | 18,0% | 16,4% |
| Ter2 |  | -0,5% | -1,2% | -1,5% | -2,4% |
| S95 |  | 0,4% | 6,0% | 0,4% | 0,6% |

MCH1: (M9 Expo/Stat) : FX49/FX48 (replicate 1)


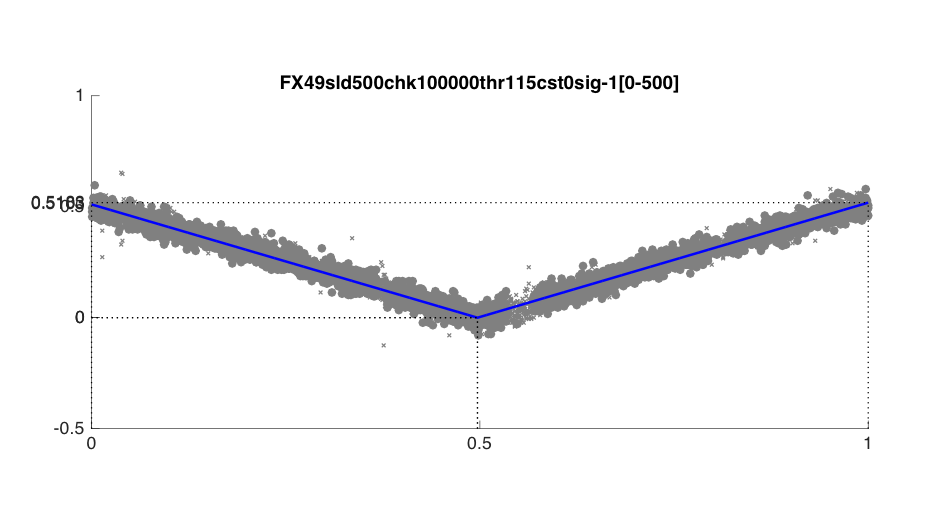


MCH1: (M9 Expo/Stat) : FX318/FX317 (replicate 2)


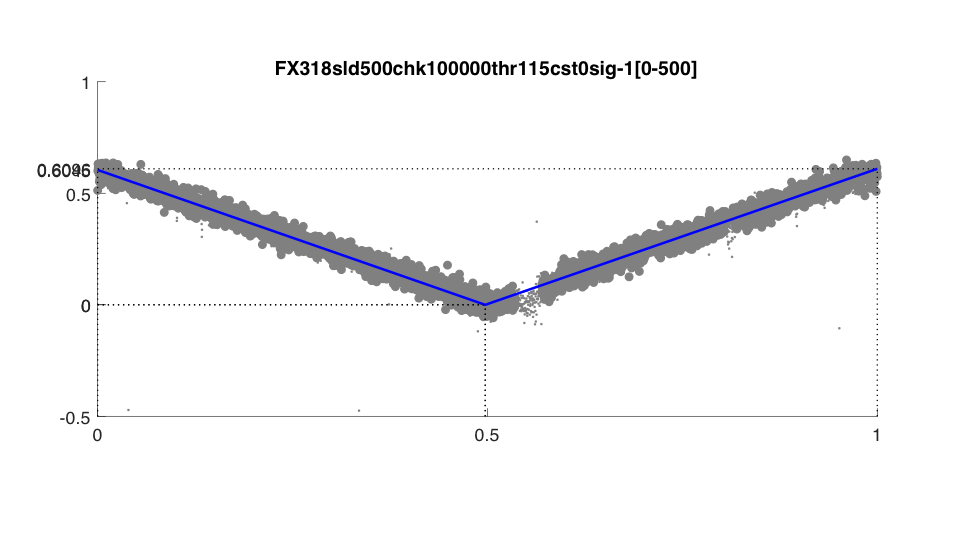


EGV369 (M9 Expo/Stat) : FX11/FX10 (replicate 1)


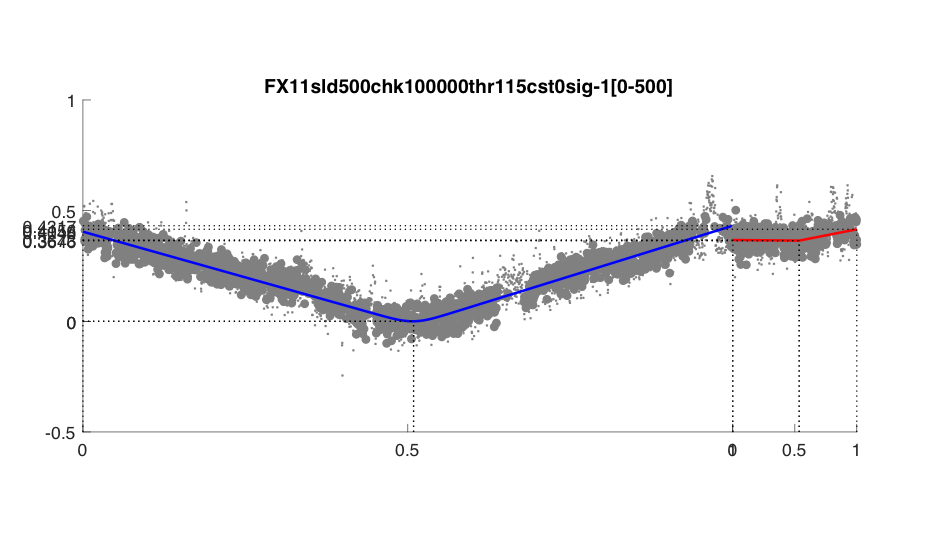


EGV369 (M9 Expo/Stat) : FX320/FX317 (replicate 2)


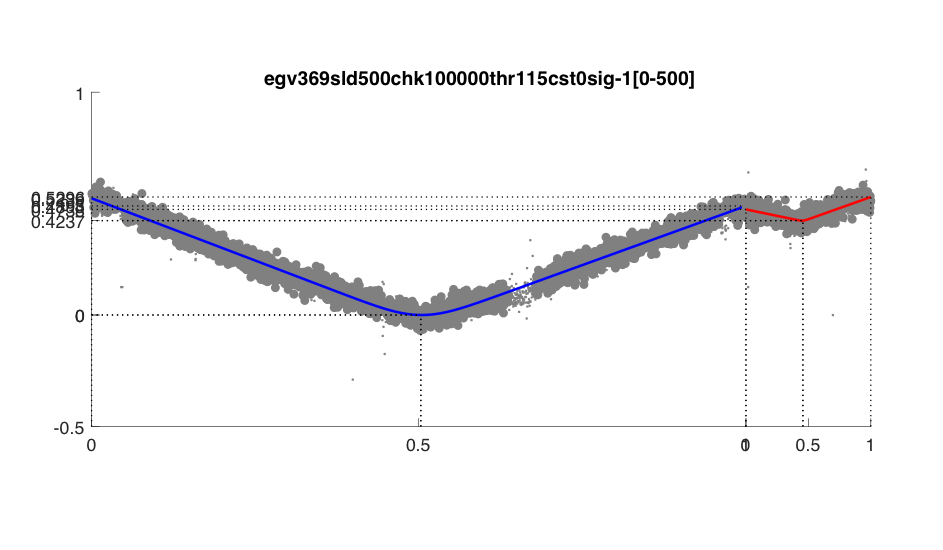
`

EGV366: (M9 Expo/Stat) : FX12/FX10 (replicate 1)


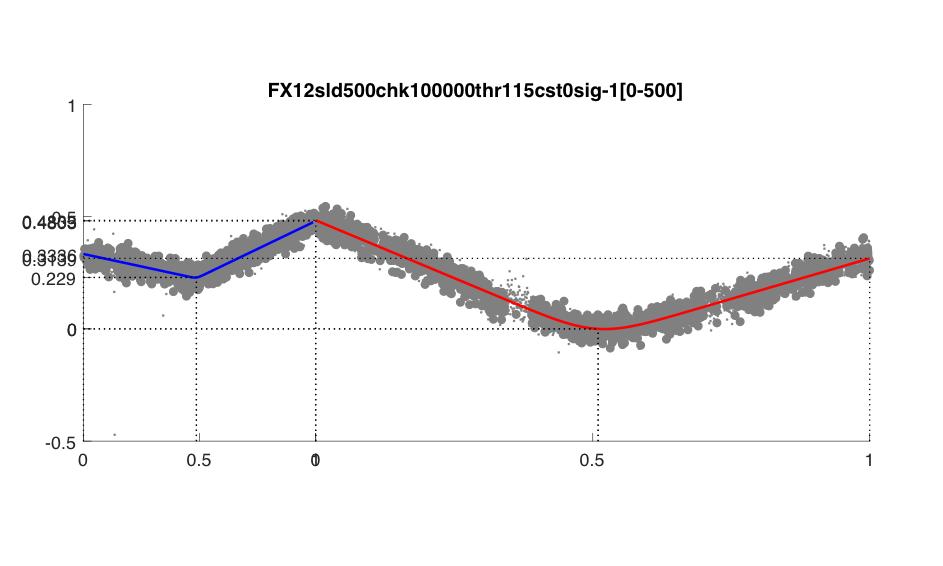


EGV366: (M9 Expo/Stat) : FX319/FX317 (replicate 2)


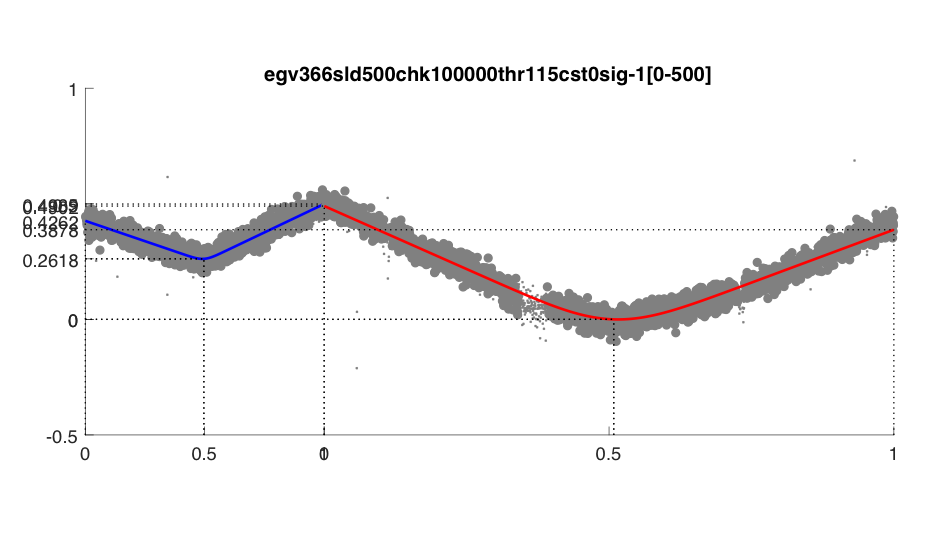


|  |  | MCH1 | | EGV366 | | EGV369 | |
| --- | --- | --- | --- | --- | --- | --- | --- |
| Sld size (kb) | | 1 | 0.5 | 1 | 0.5 | 1 | 0.5 |
| Ter1a | replicate 1 | -0,3% | -0,3% | -1,4% | -0,8% | 0,9% | 0,9% |
| S95 |  | 0,1% | 0,1% | 5,4% | 11,1% | 8,9% | 8,9% |
| Ter1b |  |  |  | 0,9% | 0,8% | 3,4% | 8,3% |
| S95 |  |  |  | 22,8% | 22,0% | 0,6% | 1,2% |
|  |  |  |  |  |  |  |  |
| Ter1a | replicate 2 | -0,3% | -0,29% | -0,4% | -0,2% | 0,3% | 0,4% |
| S95 |  | 0,1% | 0,0% | 12,4% | 16,3% | 17,5% | 16,3% |
| Ter1b |  |  |  | 0,8% | 0,7% | -4,5% | -4,1% |
| S95 |  |  |  | 30,8% | 28,1% | 30,8% | 28,1% |

**References**

1. Demarre, G. *et al.* Differential management of the replication terminus regions of the two Vibrio cholerae chromosomes during cell division. *PLoS Genet.* **10**, e1004557 (2014).

2. David, A. *et al.* The two Cis-acting sites, parS1 and oriC1, contribute to the longitudinal organisation of Vibrio cholerae chromosome I. *PLoS Genet.* **10**, e1004448 (2014).

3. Bremer, H. & Churchward, G. An examination of the Cooper-Helmstetter theory of DNA replication in bacteria and its underlying assumptions. *J. Theor. Biol.* **69**, 645–654 (1977).
